# Statistical Inference for Valued-Edge Networks: The Generalized Exponential Random Graph Model

**DOI:** 10.1371/journal.pone.0030136

**Published:** 2012-01-19

**Authors:** Bruce A. Desmarais, Skyler J. Cranmer

**Affiliations:** 1 Department of Political Science, University of Massachusetts Amherst, Amherst, Massachusetts, United States of America; 2 Department of Political Science, University of North Carolina at Chapel Hill, Chapel Hill, North Carolina, United States of America; University of Zaragoza, Spain

## Abstract

Across the sciences, the statistical analysis of networks is central to the production of knowledge on relational phenomena. Because of their ability to model the structural generation of networks based on both endogenous and exogenous factors, exponential random graph models are a ubiquitous means of analysis. However, they are limited by an inability to model networks with valued edges. We address this problem by introducing a class of generalized exponential random graph models capable of modeling networks whose edges have continuous values (bounded or unbounded), thus greatly expanding the scope of networks applied researchers can subject to statistical analysis.

## Introduction

The need to analyze networks statistically transcends disciplines that have occasion to study the relationships between units. Applications in the medical sciences [1]–[3], physics [4]–[8], computer science [9], [10], mathematics [11]–[13], the social sciences [14]–[16], and other fields examine networks that vary in size and density, over time, and have edges with values that vary from binary ties, to counts, to bounded continuous and unbounded continuous edges. An important method for statistical inference on networks is the exponential random graph model (ERGM) [17]–[19], which estimates the probability of an observed network conditional on a vector of network statistics that capture the generative structures in the network. Yet the ERGM has a major limitation: it is only defined for networks with binary ties [20], [21], thus excluding a wide range of networks with valued edges (e.g., genetic networks [22] and correlation networks [23]). We develop a class of generalized ERGMs (GERGMs) for inference on networks with continuous edge values, thus lifting the restriction of this methodology to a, possibly small, subset of networks. The form of our generalized model is similar to the ERGM in that it can be flexibly specified to cover a broad range of generative features, and our model can be estimated efficiently with a Gibbs sampler. The strengths and limitations of the ERGM are apparent from its functional form. Let 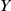 be the 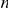-vertex network (adjacency matrix) of interest with 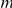 edges (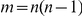 if 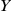 is directed and 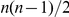 if it is undirected). 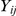 is the edge from 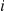 to 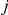. An ERGM of the network 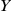 is specified as:
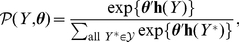
(1)where 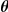 is a parameter vector, 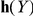 is a vector of statistics computed on the network, and the object of inference is the probability of the observed network among all possible permutations of the network given the network statistics. The 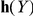 term is what gives the ERGM much of its power: this vector can contain statistics to capture the interdependence structure of connectivity in the network – statistics can be included to capture reciprocity, transitivity, cyclicality, and a wide variety of other endogenous structures – as well as the effects of exogenous covariates [24].

The challenges for modeling networks with valued edges are apparent from the specification in equation 1. The flexibility of the ERG distribution comes from the lack of constraints in specifying 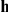; the only constraint is that 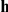 is finite when evaluated on any binary network. This assures that the denominator is a *convergent* sum, and therefore represents a proper normalizing constant for the distribution of networks. However, this convergence is not assured whenever 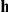 is finite if the support of 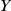 is infinite, as it is with any network with continuous-valued edges. The model we derive retains the flexibility of 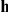 within a framework that assures a proper probability distribution for 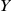 when 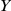 has continuous edges.

## Methods

The major strength of the ERGM is that the vector of statistics on the network, 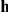, can be specified to represent many forms of dependence among the elements of 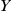, including transitivity (i.e., clustering), popularity, and reciprocity. Because these same dependence features characterize valued networks [20], [21] and can be of theoretical import [15], we seek a generalization of the ERGM that maintains the flexibility of the set of network statistics, 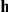, while moving away from the limitations inherent in the denominator of the ERGM. We see the analytic challenge of defining an ERGM-like model for valued networks as a three-part problem: deriving a distributional family that is (1) guaranteed to have a convergent normalizing constant, (2) incorporates dependence functions into the distribution as flexibly as does the ERGM, and (3) is easily adapted to accommodate a variety of edge types (e.g., bounded, unbounded, strictly non-negative). In this section, we introduce a method of constructing joint *continuous* distributions on networks that permit the representation of dependence features among the elements of 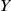 through a set of statistics on the network, 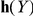. This *generalized exponential random graph model* (GERGM) can be used when edges are continuous and unbounded, bounded from above, bounded from below, or bounded above and below; thus greatly increasing the scope of networks it can analyze compared to the ERGM.

### The Generalized ERGM (GERGM)

There are two specification steps in our approach to generalized ERGMs (GERGMs): first, we specify a tractable joint distribution that captures the dependencies of interest on a restricted network, 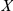, and then we transform 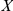 onto the support of 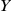. In so doing, we produce a probability model for 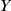. To illustrate these steps, begin with consideration of the restricted valued network 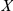, which has the same vertices as 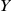, but edge values that are continuous and bounded between zero and one (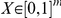).

Our first specification step involves defining a set of network statistics, 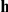, to capture endogenous effects and exogenous covariates, and defining a probability distribution for the restricted valued network 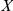. We define a probability distribution for 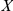 by adapting the ERGM formula presented in equation 1 to address a 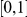 bounded network and assure a convergent sum in the denominator:
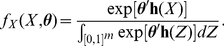
(2)In equation 2, 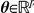 remains the parameter vector and 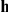: 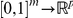, is formulated to represent joint features of 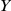 in the distribution of 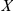. The statistics 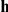 are guaranteed to be finite on 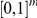 and each 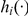 is a statistic that captures the generative structure of the network by summing over subgraph products such that for every 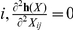. This is a flexible specification because many dependence relationships can be captured by summing products over subgraphs of the network, particularly when the edges are in the unit interval [21]. For instance, networks generated by a highly reciprocal process are likely to exhibit high values of 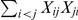, and those in which connections gravitate toward high-degree vertices exhibit high values of 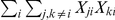 (i.e., “two-stars,” [25]).

An important property of the distribution we have specified for the restricted valued network, 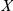, is that when there are no dependencies in the network, 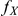 is an appropriate model for independent uniform random variables. That is to say, if we have correctly specified the set of network statistics and 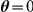, then 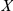 has no dependencies. Since 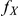 is the joint distribution of the quantiles of 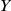, and a joint uniform distribution is the joint distribution of the quantiles of independent random variables [26], 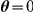 implies independence among the edges in 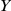. This is convenient because it implies that there need not be any dependencies in the network to use the GERGM.

In our second specification step, we transform the restricted valued network 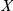 onto the support of the network of interest 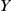. We do so by applying parameterized, one-to-one, monotone increasing transformations, which we denote 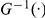, to the 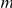 edges of the restricted network. Specifically, we specify 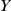 as
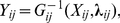
(3)where 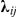 parameterizes the transformation to capture marginal features of 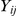. Equation 3 shows that we can define each edge, 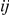, in the network of interest (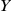) as a parameterized transformation of the same 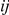 edge in the restricted network 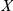. An interesting case of transforming 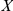 is when the edges of 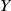 are bounded from below at 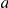 and above at 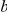. In this case, the transformation 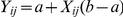 is a natural choice. This illustrates that the GERGM can be used to model networks of correlation coefficients, which have been of great interest recently [27]–[29].

Given this transformation of the restricted network, we derive a specification for the GERGM that allows us to keep the basic structure and strength of the ERGM: the 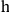 vector is now specified on a transformation of the network rather than the network in its observed form, but it maintains all the flexibility that makes the ERGM powerful. Because 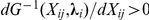, the properties of multivariate transformations [30] imply that the distribution of 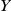 is 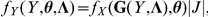 where the Jacobian matrix, 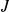, is the matrix of first partial derivatives. Since 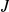 is a diagonal matrix, we may write the GERGM as
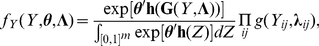
(4)where the model parameters 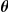 and the transformation parameters 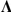 must both be estimated.

An elegant feature of this formulation is that it may be specified to reduce to well known regression models for independent data when the network is free of dependencies. Specifically, we may specify 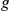 as a probability density function (i.e., 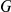 is a CDF, and 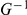 an inverse CDF) parameterized to match the support of 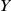 and capture features of 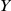 such as location, scale, and dependence on covariates. When 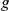 is specified as such, the distribution for 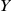 contains many common models for independent and identically distributed variables as special cases when 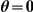. For instance, if 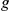 is a Gaussian PDF with constant variance and the mean dependent on a vector of covariates, the model reduces to that assumed in linear regression. This is a useful feature of the model because researchers may doubt the role of network dependencies in their data, but be uncomfortable applying a model that assumes no dependencies and is incapable of modeling them (e.g., regression). In such a case, the researcher may apply a GERGM and, if there are no dependencies, the parameters 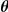 that capture network dependencies will be zero and the parameters returned for exogenous covariates will be identical to those a regression would have produced.

A further feature of the GERGM for researchers unsure of whether to include some subset of their effects, be they endogenous dependencies or exogenous covariates, is that the GERGM allows hypothesis tests for block restrictions. As such, a researcher may apply tests, such as the likelihood ratio or Wald tests, to test the assumption that the edges of 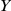 are independent conditional upon 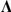.

The specification of dependencies in a quantile network is standard across different edge-types, because the support of the joint quantiles is always a unit hypercube. However, the specification of 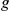 will vary substantially based upon the marginal characteristics of 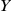. A few general features to consider when selecting 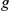 are (1) the support of 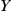, (2) the notable characteristics of the moments of 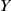, and (3) the dependence of 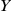 upon covariate information. It is advisable to select 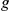 such that the support of 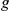 is equal to the possible values that could be observed for 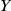. For instance, if the edge values are strictly positive (e.g., monetary exchange), a Weibull distribution would be a feasible choice. Once a class of 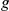
's with appropriate support is identified, it is then important to consider other relevant marginal features of 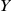 – such as skewness, kurtosis, or multimodality – and be sure to choose a 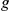 that is flexible enough to represent those marginal features. Lastly, it might be the case that marginal characteristics of 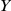 vary based on some covariate information. It is important to parameterize 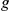 such that these dependencies can be accurately represented. One beneficial feature of our two-stage derivation of the GERGM is that the extensive literature on fitting flexible parametric models to independent observations can inform choices for 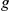 (e.g., [31]).

It is also important to note that inferences about network dependencies will depend upon the specification of 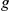. The network dependencies are estimated on the joint quantiles with respect to 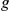. Thus, changing 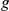 alters the joint quantiles of 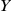 with respect to 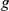 and effectively changes the network within which the dependencies are estimated. In this sense, we do not expect that inferences with respect to 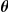 will be robust to substantially different choices of 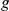. It is therefore important to consider and compare feasible alternatives for 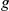. Typically, evaluating the robustness of a particular model to alternative specifications of 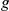 will not be especially difficult because nested alternatives can be compared using Wald tests on the parameter restrictions. Simulation based model-fit metrics, such as those computed in our application below, could also be used to compare alternative formulations of 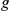. An important topic for future research would address model comparison and selection within the GERGM framework.

**Figure 1:**
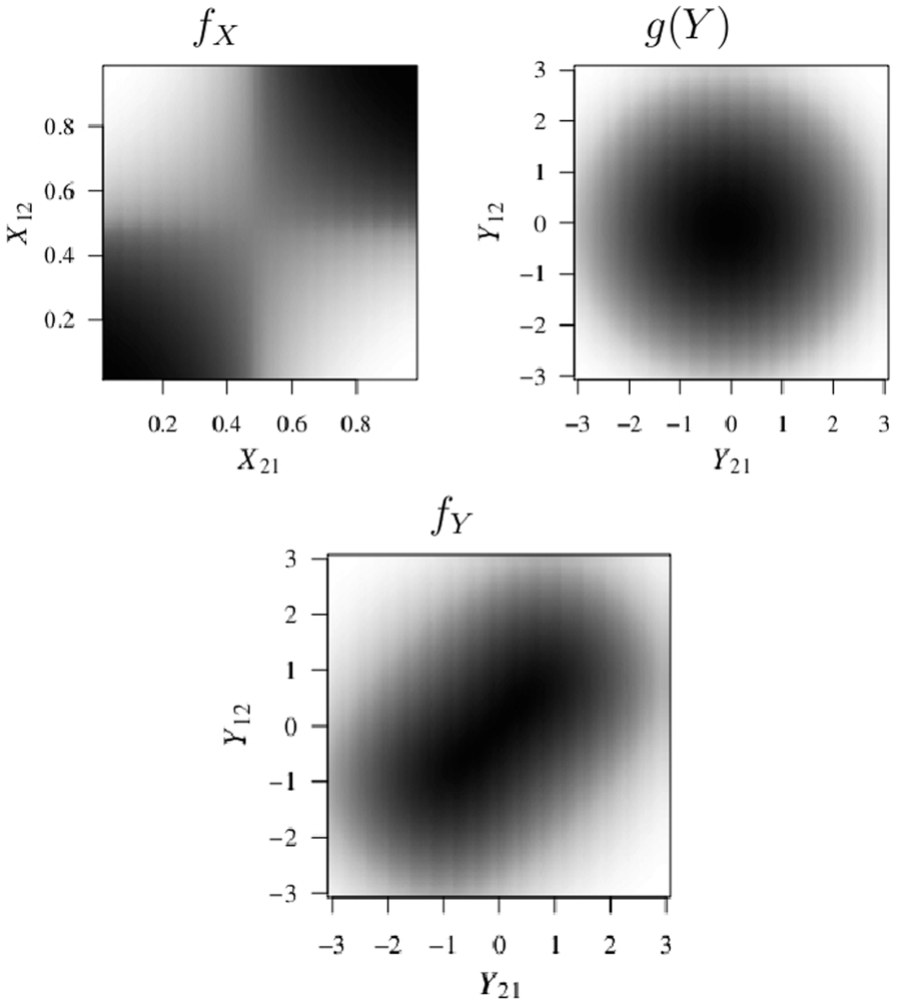
Bivariate distributions for edges in a two-vertex di-graph. (**c**) The darker the shading, the higher the relative likelihood of a point. In this example, 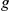 is the standard normal PDF (b), and 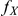 (a) is defined by 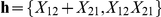, and 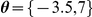, representing negative density and positive reciprocity effects.

Interpretation of the GERGM coefficients is relatively straight forward and we give an extensive example when we present our application. We note here however that, when 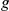 is a PDF, 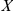 is the random variable drawn from the joint distribution of the quantiles of 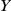. Therefore, the vectors 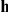 and 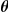 characterize the dependencies among the quantiles of 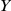. In this way, our method closely resembles the process of constructing joint distributions with copula functions [26]. To illustrate the process of specifying a GERGM, it is useful to consider a generic small-scale model. A simple example of deriving a joint distribution through the combination of 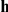 and 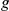 is illustrated in Figure 1, which presents the distributions of 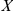 and 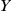 for a directed network with two vertices exhibiting a high degree of reciprocity.

### Alternative Formulations

Our approach to the generalized ERGM is not the only means by which the ERGM can be extended to model valued-edge networks, though we believe it is a particularly flexible one. Krivitsky [32] has proposed an alternative framework for such an extension, which takes a substantially different approach to the problem than we do. As noted above, one of the major challenges to deriving an ERGM for a network with infinite support is that of assuring that the sum or integral over the probability mass or density function is convergent. We assure this by defining the exponential family graphical model on the restricted quantile network. This permits free reign in the specification of dependence functions 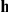. The only requirement is that the functions be finite-valued. The approach to assuring a convergent sum/integral, and thus a proper probability distribution, taken by Krivitsky [32] is more flexible than ours, yet imposes more constraints on the definition of 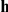. The extension of the ERGM proposed by Krivitsky [32] is given by
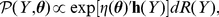
(5)where 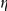 maps 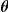 to canonical parameters and 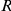 is a ‘reference measure’ that assures
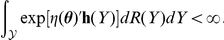
For a given reference measure, 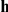 must be carefully specified so as to be dominated by 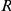.

It is not apparent that either approach is globally preferable. Our approach permits substantially greater flexibility in specifying 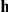, since there is no need to check for convergence given a particular specification of 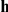. However, we restrict the specification of dependence to occur within the joint quantile network. Indeed, we view the necessity that the dependencies be estimated in the joint quantile network as the primary limitation of our formulation of the GERGM. The class of models proposed by Krivitsky [32], in contrast, permits dependence to be represented in the natural support of 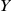. However, our framework offers a more direct relationship between the GERGM and common independence models than that proposed by Krivitsky [32]. For instance, in the Poisson ERGM proposed by Krivitsky [32], independence among the edges in the network does not assure that the edges are marginally Poisson distributed. In our formulation of the GERGM, when the edges are independent, the model is guaranteed to reduce to the marginal model used to specify 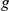. Ultimately however, which model is more appropriate will depend on the particular application.

### Estimation

Estimation of the parameters in the model is a non-trivial task. The greatest challenge in estimating 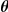 and 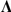 in equation 4 is that the integral in the denominator is typically intractable. Because of the polynomial structure of 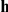, and the fact that the variables of integration are bounded, we know that the integral is both positive and finite, meaning 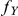 is a proper joint distribution. However, inference requires the approximation of the denominator. We develop a Markov chain Monte Carlo maximum likelihood estimation (MCMC-MLE) [33] method for estimating the parameters.

In order to approximate the denominator in equation 4, we sample from 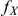 using a Gibbs Sampler. To do so, we require the conditional distribution of 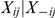. To simplify the notation, let 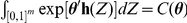. The conditional distribution (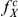) is given by
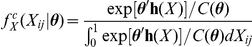


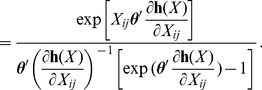
(6)We may then draw from the conditional distribution in equation 6 using the inverse CDF method. If 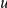 is a uniform (0,1) random variable, then
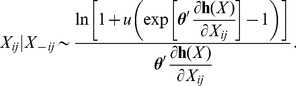
(7)When 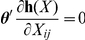 the conditional density given in equation 6 is undefined. However, in this case, each point in the unit interval is equally likely and the conditional distribution of 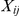 is uniform (0,1).

In order to estimate 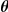 and 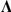, we maximize 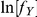:
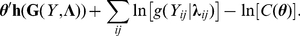
(8)Our algorithm iteratively proceeds by maximum likelihood estimation of 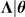 and MCMC-MLE of 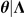 until convergence. We derive an approximation to the asymptotic variance-covariance matrix by the inverse of the negative Hessian matrix at the last iteration.

Consider first the maximum likelihood estimation of 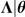. Because 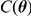 does not depend on 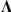, maximum likelihood estimation of 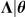 reduces to
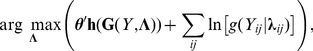
(9)a function easy to maximize using a hill-climbing algorithm.

**Figure 2:**
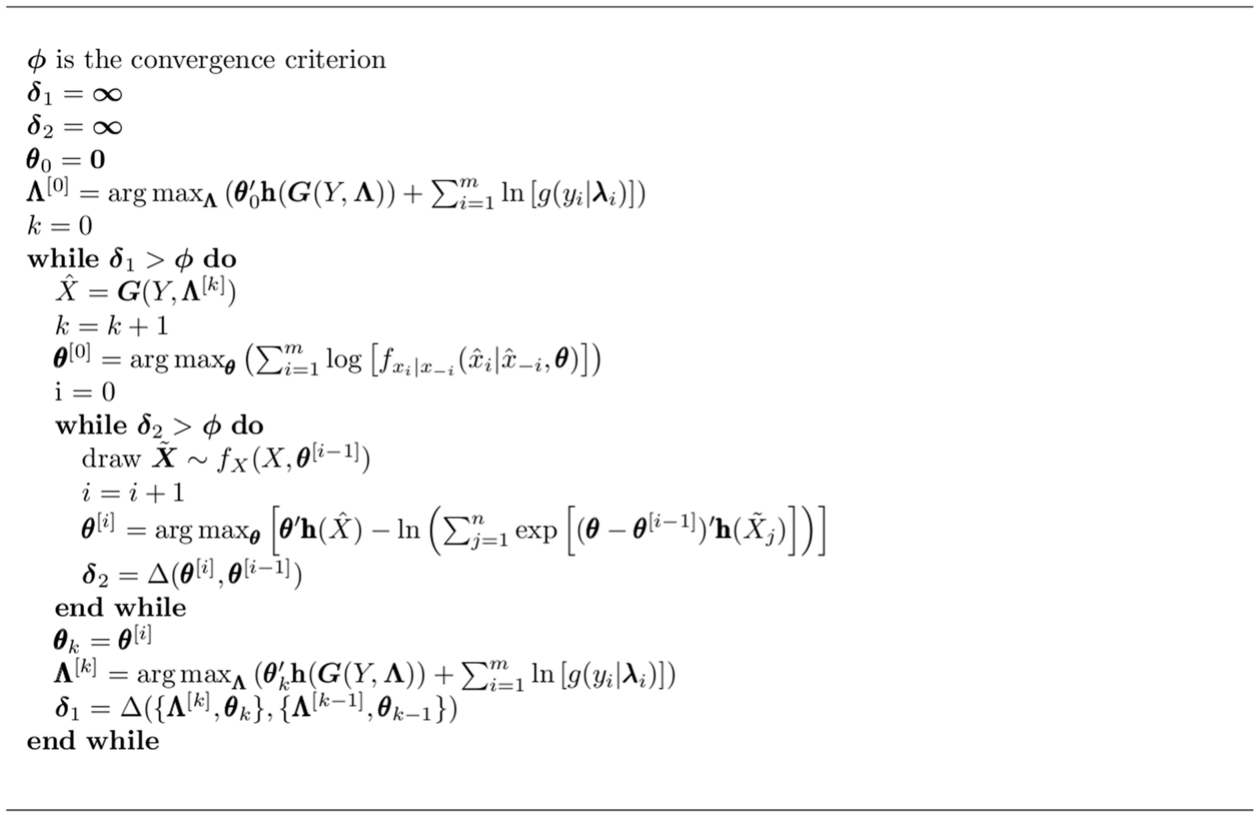
Estimation by iterative MLE-MCMC-MLE.

The estimation of 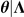 is more involved. Let 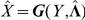 be the estimate of the restricted (quantile) network given the current estimate of the transformation parameters. The second term in equation 8 does not depend on 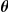, so to estimate 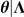 we find
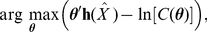
(10)which requires an approximation of 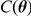. We approximate 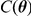 using MCMC-MLE; an iterative method itself. Let 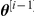 be the previous estimate of 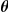, and 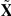 be a sample of 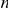 networks drawn from 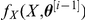. Then, an approximation to 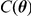 is given by
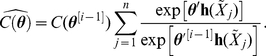
(11)This requires a starting value for 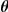. In simulation experiments, we have found the pseudolikelihood estimate (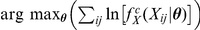) to be effective in providing starting values for 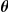 (i.e., 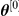). Pseudocode for the algorithm is given in Figure 2.

### Challenges in Estimation and Specification

The joint distribution 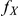 in equation 2 is a linear exponential family multivariate distribution in that 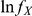 is proportional to a linear combination of the parameters 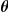 and sufficient statistics 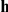 [34]. Focusing specifically on ERGMs, there is a burgeoning literature on obstacles to specification and approximate maximum likelihood estimation with multivariate discrete exponential family distributions [35]–[37]. There are two related problems that have motivated this literature: (1) the existence and uniqueness of of the MCMC-MLE, and (2) the degeneracy of the ERG distribution.

To estimate the model by MCMC-MLE, we maximize the approximate likelihood function with respect to 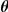, such that a sample of networks 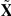 is used to approximate the likelihood function. The sample is drawn from a distribution parameterized with the same network statistics 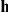 and a previous estimate or starting value for the parameter 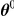. The performance of this optimization method depends heavily upon the sample 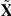, and thus upon 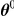. Specifically, a value of 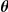 that maximizes the approximated likelihood exists and is unique if and only if the values of the network statistics computed on the observed network (i.e., 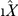) are within the 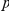-dimensional convex hull of the network statistics computed on the sample of networks. In application, this requires that 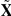 be drawn from a distribution that generates networks similar to 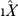. Heuristically, we would expect that setting 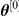 close to the true maximizer of the likelihood function would be sufficient. However, this is not the case, which brings us to the second challenge.

The problem of degeneracy in discrete exponential families adds substantial complication to the specification, estimation and simulation of ERG distributions. Discrete ERG distributions that are degenerate tend, in Markov Chain simulation, toward either the completely full graph in which all edges are at their maximum value or the completely empty graph in which all edges are at their minimum value [36]. This means that either extremely dense or extremely sparse networks have high probability in a degenerate ERG distribution. This creates two complications in application. First, degenerate ERGMs are poor models for most empirically observed networks, meaning that it is generally unacceptable to arrive at a degenerate ERGM in training a model for an observed network [36]. Second, degeneracy of the approximating distribution in the iterations of MCMC-MLE can cause the convex hull of the statistics computed on the sample of approximating networks to be far from the statistics computed on the observed network, causing the algorithm to break down [36]. Adding to the challenges posed by degeneracy, for a given model and network size, there may be only a very small and nonlinear region in the parameter space that leads to non-degenerate ERG distributions [37], which complicates the selection of starting values and the iterative search of the parameter space.

There are two complimentary approaches to combating the problem of degeneracy in ERGMs: using specifications that are less prone to degeneracy and checking a given estimated model for degeneracy. First, the degree to which a particular ERGM is prone to degeneracy depends substantially on the specification of the model [37]. Classic ERGM specifications used counts of sub-graphs that measure local dependence structures as network statistics (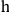). For example, to measure transitivity (i.e., whether a friend of a friend is a friend), classically specified ERGMs used counts of the number of triangles in the network. Classically specified ERGMs are known as Markov Graphs [38]. To minimize degeneracy problems, Snijders, Pattison, Robins and Handcock [39] proposed a set of specifications of the ERGM that are substantially less prone to degeneracy than Markov Graphs. This is a useful approach to the problem because use of these specifications reduces the probability that model selection/specification will be complicated by degeneracy.

Second, one can directly check whether a given ERGM is degenerate. This is accomplished in a straightforward manner by simulating a large number of networks using MCMC and checking whether (a) the simulated network statistics are similar to the observed values and (b) whether the Markov Chain is tending toward the full or empty graph [40]. This is a powerful approach to diagnosing degeneracy because it can be applied to any ERGM specification. Indeed, regardless of the specification used, it is important to diagnose whether an estimated model is degenerate because even degeneracy-resistant specifications do not guarantee non-degeneracy.

Because the GERGM is based on a continuous exponential family and is applicable to a wide array of edge types, it is not clear that the statistics proposed by Snijders, Pattison, Robins and Handcock [39] can be easily adapted to the GERGM framework. Thus, though outside of the scope of the current research, future work should focus on developing specifications of the GERGM that are resistant to degeneracy.

Fortunately, however, it is straightforward to apply the same MCMC methods used in estimating the model to determine whether a particular GERGM is degenerate. We take a two-pronged approach to checking for degeneracy. First, we check whether the average edge value in the simulated networks is closer to zero or one than to the mean of the network used to estimate the model. This can be accomplished through the use of trace plots (a line-plot of connecting mean edge values over many iterations of the chain) and/or running mean plots (a plot to examine the stability of the mean edge value over a large number of iterations of the chain); though trace plots may be better suited to this purpose than running mean plots because they show every mean value. Second, once we are satisfied that the means in the simulations are far from degenerate values, we use standard MCMC diagnostic tools to test for non-convergence of the Markov chain. The Geweke and Gelman-Rubin diagnostics lend themselves particularly well to this purpose. As with all convergence diagnostics, the Geweke and Gelman-Rubin tests are tests of non-convergence that assume the convergence of the chain as the null hypothesis; accordingly satisfying these diagnostics does not assure convergence, but provides the best indication of convergence possible given that analytical proofs of convergence are not possible.

The Geweke diagnostic [41] is a time-series diagnostic based on a comparison of two non-overlapping windows of the Markov chain, one earlier in the series and one later. The Geweke diagnostic is specified as
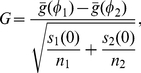
(12)where 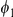 and 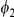 are non-overlapping subsets of the Markov chain of length 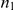 and 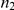 respectively, the 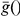 function is typically the mean, and 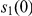 and 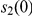 are the symmetric spectral density functions [42]. Because the Geweke diagnostic follows a standard normal distribution, one typically takes values greater in absolute value than 2 to indicate non-convergence.

The Gelman-Rubin diagnostic [43] examines the convergence of multiple Markov chains begun from several overdispersed starting points by estimating the factor by which the distribution of parameter 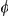, at any point in the Markov chain, is expected to shrink under continued simulation. For 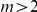 Markov chains of length 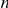, the within and between chain variances are respectively
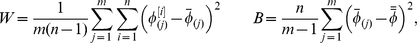
(13)where 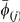 indicates the mean for the 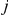
th chain, and 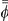 indicates the grand mean [42]. The total variance may then be calculated as 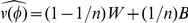 and the shrink factor is computed
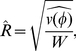
(14)where values departing significantly from 1 indicate non-convergence [42], [44].

If we can satisfy ourselves that the running mean of network edge values is non-degenerate and that the Markov chains have converged, we will have satisfied the strongest possible criteria for claiming non-degeneracy of the GERGM model.

## Results

We illustrate important features of the GERGM and demonstrate its efficacy by applying it to a real network: the network of domestic migration in the United States. Our aim in this application is primarily pedagogical, and so we devote more attention to the choices made as part of the modeling process and alternative ways to interpret our results than is typical of applications whose primary purpose is substantive discovery.

Interstate migration flows in the U.S., the flow of citizens from one state to another, do much to shape the demographic, political, and economic makeup of the country. Migration flows have implications for local financial markets [45] and are an important determinant of stress on public infrastructure [46]. What is more, consumer-voters are thought to relocate to states that better match their preferences [47] and, perhaps as an effect, migration can shape the political climates of the states [48]. Migration flows naturally form a directed and valued network because each state (vertex) sends a certain number of its citizens to every other state (outbound edges), and receives a certain number of citizens from every other state (inbound edges). Despite some recent interest in modeling migration as a network phenomenon [49]–[51], there is little work in this area and the literatures in policy/political science and demography have not been well integrated. Our aim is to demonstrate the GERGM on interstate migration flows while incorporating factors from both literatures.

In contrast to previous studies, we focus on the change in the directional interstate migration flow from one year to the next. Migration flows are fairly persistent over time, and the ability to predict this year's flow based on the previous year's may mask an important type of predictive deficiency in a statistical model. Substantial change in the migration in and out of a state are of interest because they can cause disruptions to local economies and exert unexpected stresses on infrastructure. Specifically, we model the change in interstate migration flows from 2006 to 2007, in the 50 states, Washington D.C., and Puerto Rico. The edge from state 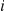 to state 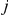 is the difference between the number of people who migrated from 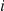 to 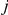 in 2007 and the number who migrated from 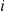 to 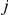 in 2006. These data allow us to consider the GERGM in the context of a valued network requiring transformation away from the restricted valued network onto a continuous unbounded support with exogenous covariates and endogenous parameters, thus making full use of the GERGM's flexibility.

**Figure 3:**
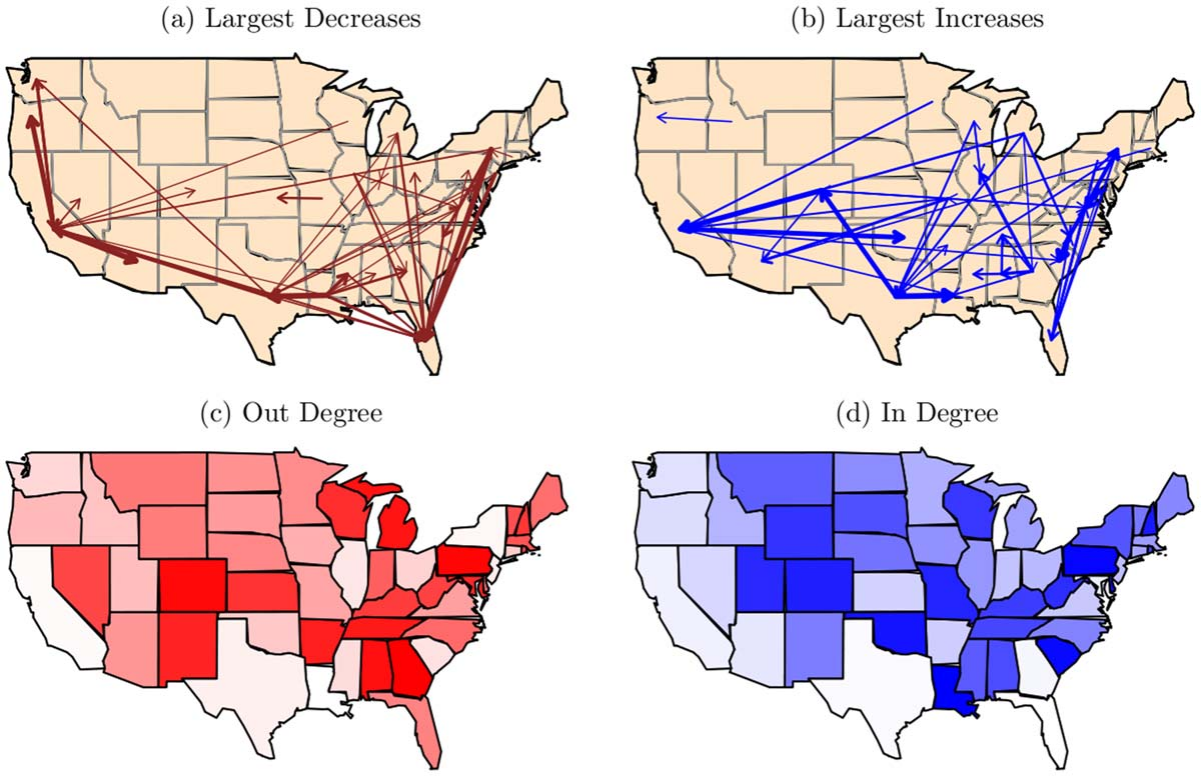
The increases and decreases in year-to-year migration. The upper-left and upper-right plots respectively show the largest 5% of decreases and increases from one state to another; the width of the line corresponds to the magnitude of the exodus. The lower-left and lower-right plots display the states with the highest total number of citizens leaving and the highest total number of citizens arriving respectively. These data are available at http://www.census.gov/population/www/socdemo/state-to-state.html.

To gain intuition about the network under consideration, we present the largest increasing and decreasing edges and vertices in Figure 3.

There are three broad choices we face in specifying the model for the network of migration change: the selection of the distribution family for 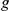, the covariates that condition the location of 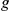, and the statistics that comprise 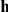.

With respect to the distribution of 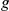, one distinct feature of the data that we need to accommodate is the thickness of the tails. The empirical kurtosis of the edges is 637, compared to the normal distribution's kurtosis of 3. As such, we use the location-scale Cauchy distribution [52]. The PDF of the Cauchy is
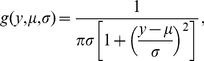
(15)where 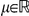 is the location parameter (i.e., the median), and 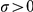 is the scale parameter. The location parameter for the edge from 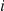 to 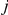 depends on a vector of covariates 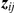 via regression parameters 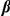, such that 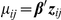. Under the restriction that there are no dependencies in the network (i.e. 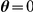), our model of change in migration flows reduces to the Cauchy regression model (CRM) [52]. Thus, we denote the model without network effects by CRM.

We draw directly from the literature on interstate migration in selecting the covariates. Specifically, we include the covariates that [49] finds to be statistically significant determinants of migration flows. These include the population, unemployment rate, per-capita income, and average January temperature of both the sending and receiving states. Since we are modeling change in and not the level of migration, each covariate is included as the change in the respective covariate value from 2005 to 2006. For instance *Unemployment Sender* (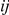) is the difference between state 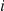
's unemployment rate in 2006 and state 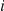
's unemployment rate in 2005.

We complete our specification by considering which endogenous dependence terms to include in the model. We include five terms to capture the endogenous generative structure of the network. The first endogenous effect we include is *transitive triads*, which will account for any unmodeled clustering in the network (e.g., migration in clusters of agricultural or coastal states). The transitive triads term is defined as
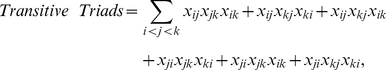
(16)where the six additive terms capture every possible combination of directed edges between three vertices: 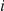, 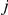, and 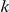. The second dependence term is *reciprocity*, which will account for any tendency towards dyadic exchange of migration flows (i.e., states trading migrants at similar levels). The reciprocity term is specified as
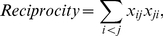
(17)which captures the tendency of 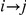 and 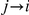 edges to co-occur. The third term we include is *cyclic triads*, which will model the tendency towards generalized reciprocity in the network – the degree to which total flows to and from a state are correlated [53]. This term is specified as
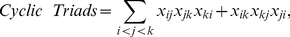
(18)and captures reciprocal effects that flow through a third state. The last two terms are closely related: *in-two-stars* and *out-two-stars*. These terms account for any unmodeled features of states that motivate flows to and from states respectively. The terms are specified as
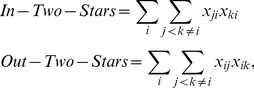
(19)and capture the tendency for other states, 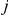 and 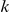, to send migrants to state 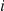, and for state 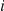 to send migrants to 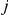 and 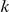 respectively.

**Figure 4:**
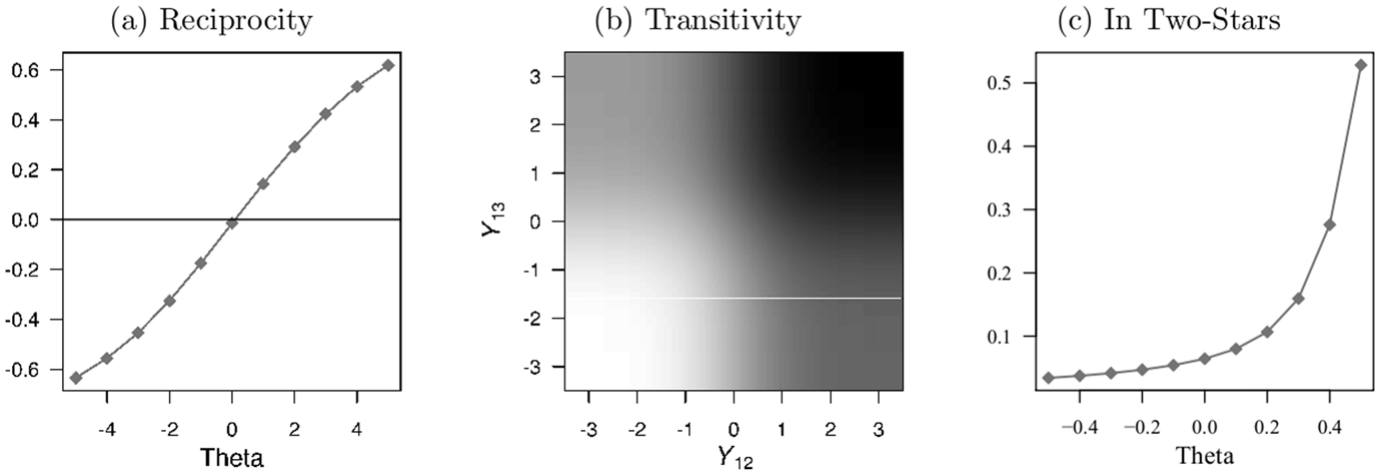
Dependence statistics in a 25 vertex network 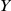
 **with a standard normal** 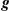 **.** The Y-axis in (a) is the Pearson's correlation coefficient between edges in a dyad. The transitivity graphic in (b) is shaded to reflect the mean value of 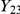, with darker values indicating higher values. The parameter value is set to 1. The Y-axis in plot (c) depicts the variance in the in-degrees across vertices.

The substantive interpretations of these statistics are illustrated in Figure 4. The plots present relevant quantities, computed on networks simulated using the network statistics discussed above, plotted against values of the parameter for the respective statistic. Quantities are derived as the average over 1,000 simulated networks. The 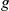 in this artificial example is a standard normal PDF, but any appropriate PDF could be used. All of the network statistics specified on 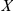 result in properties of 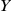 that reflect the respective dependency. As the reciprocity parameter increases, the correlation between the values of 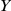 in a dyad increase. As the in two-star parameter increases, the variance in in-degree increases. Also, when the transitivity parameter is positive, the expected value of the third edge in a transitive triad increases with the values of the other two edges in the triangle. It is important to note that these are not the only conceivable measures of their respective network dependence properties. For example, see [54] and [55] for alternative measures of transitivity in valued networks. We utilize these measures because they are consistent with the product specification used in the ERGM framework, but other network statistics can be easily incorporated into the GERGM.

**Figure 5:**
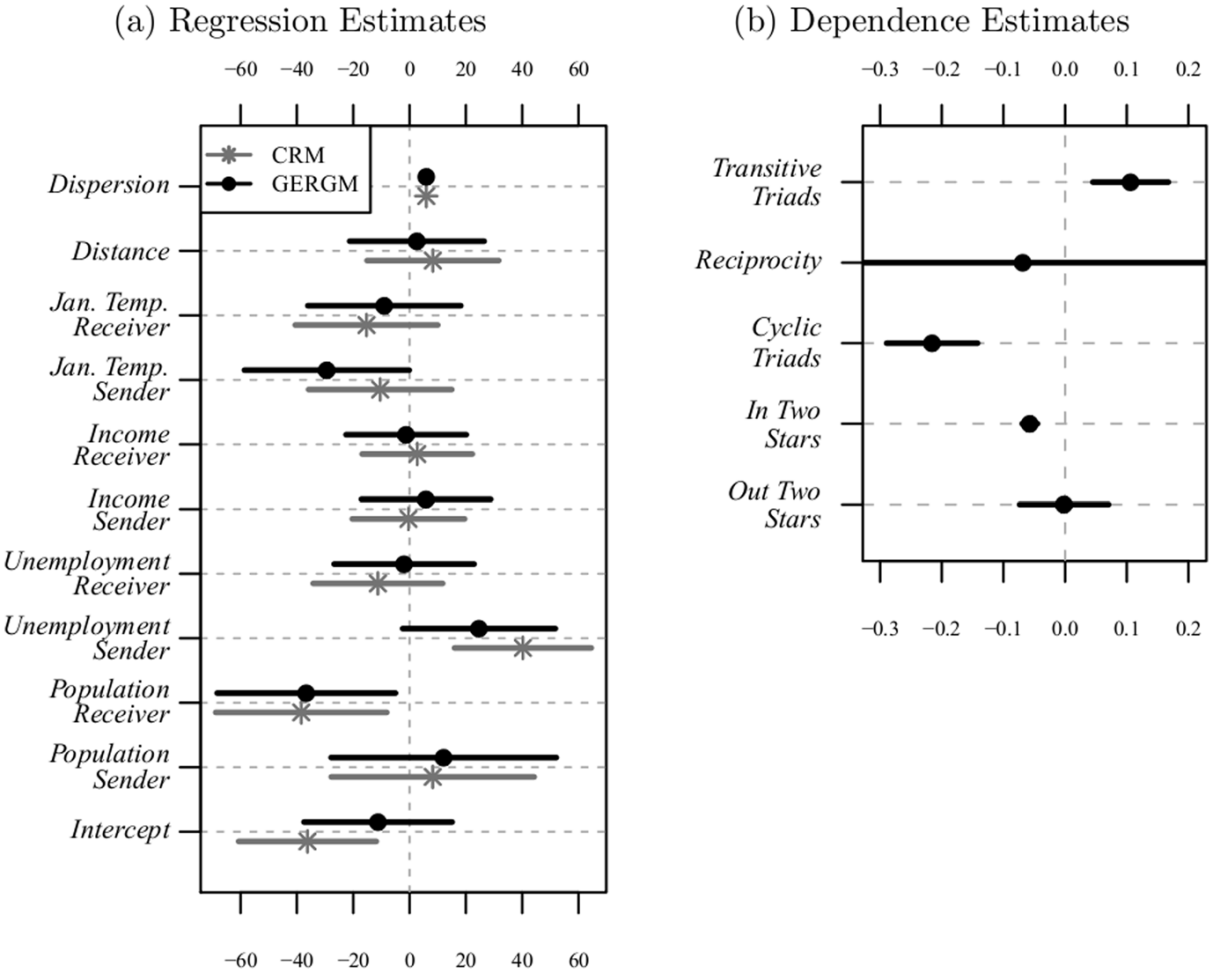
Estimates of the parameters for covariates (cell a) and dependence terms (cell b). The coefficients are depicted as points whose values are captured by their location on the x-axis. The bars spanning from each point are 95% confidence intervals based on 5,000 draws for three iterations used in the MCMC-MLE. Confidence intervals not including zero are statistically significant at the traditional 0.05 level. Points and lines in black refer to our Cauchy GERGM, those in grey refer to the CRM.

**Figure 6:**
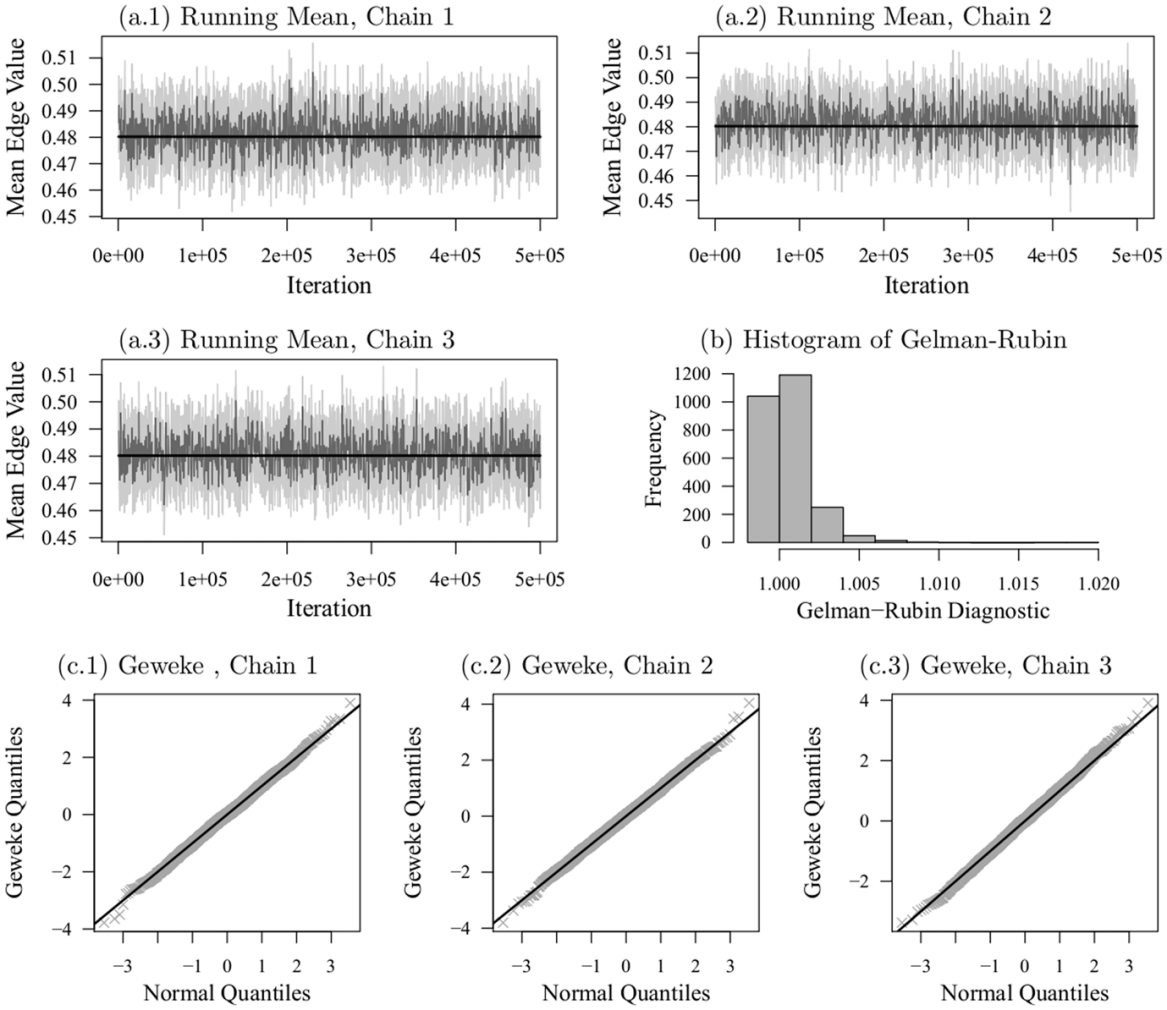
MCMC-based Degeneracy Diagnostics. Plots depict diagnostics for the GERGM results reported in Figure 5. Diagnostics are computed on three Markov Chains of 500,000 networks each, constructed via 500,000 iterations of a Gibbs sampler in which a complete network is drawn in each iteration. Each chain is started at a network with highly dispersed start values drawn from a U-shaped distribution on the unit interval, followed by a burn-in of 10,000 iterations. Panels (a.1)–(a.3) give the trace plots of the chains by iteration. The dark gray lines track the mean edge value and the light gray lines track the 95% confidence interval around the mean. Panel (b) gives the histogram of the Gelman-Rubin diagnostic of whether the three chains converged to the same stationary distribution, over all 2,550 directed edges in the migration network. Panels (c.1)–(c.3) give normal quantile plots, which compare the distribution of the Geweke time serial convergence diagnostic over the edges within each chain to the null standard normal distribution (i.e., the distribution implied by the null hypothesis of a chain in convergence). Note: the R package coda [57] was used to compute the Geweke and Gelman-Rubin diagnostics.

Figure 5 shows the estimates from our GERGM as well as estimates from the CRM. As we consider the results, it is important to assess whether the estimated GERGM is degenerate. Our GERGM shows no indication of degeneracy. We simulate networks from the GERGM via three independent Markov chains of 500,000 iterations, using a Gibbs sampler that draws a conditional edge for each directed pair of vertices in each iteration, using the conditional distribution in equation 6. Our approach includes much more simulation within each iteration, as compared to the standard Metropolis-Hastings approach to simulating from ERGM, in which one edge is re-drawn in each iteration [35]. We see, in Figure 6, that (a) the mean edge value is far from zero or one, and varies around the mean of the observed network, and (b) there is no evidence of non-convergence given by the Geweke and Gelman-Rubin convergence diagnostics. Under the null hypothesis of convergence (i.e., no difference in the means at the beginning of the chain and the end of the chain), the Geweke diagnostic has a standard normal distribution [41]. The normal quantile plots in panels (c.1)–(c.3) of figure 6 show that the Geweke statistics computed on our Markov chains are distributed very close to a standard normal, which is consistent with the null hypothesis of convergence. Also, none of the Gelman-Rubin diagnostic statistics, depicted in panel (b), are at or above 1.1 – the level typically taken to indicate non-convergence across multiple chains [56].

A Wald test suggests the restriction of the dependence terms to zero, a restriction the regression model *must* make because it cannot accommodate dependence terms, is inappropriate and that the GERGM provides a better fit to the data (Wald statistic
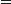
119.19 on 5 degrees of freedom, statistically significant at the 0.001 level). The statistically significant effects for the dependence parameters indicate that (a) there are clustering effects in the network, (b) migration to states repels further migration, and (c) increases in migration flows from a state are not offset by increases in flows to that state. We also find a decrease in the number of people leaving warm states, a decrease in migration to states that experienced a substantial increase in population in the previous year, and evidence of an increase in migration away from states experiencing increases in unemployment.

**Figure 7:**
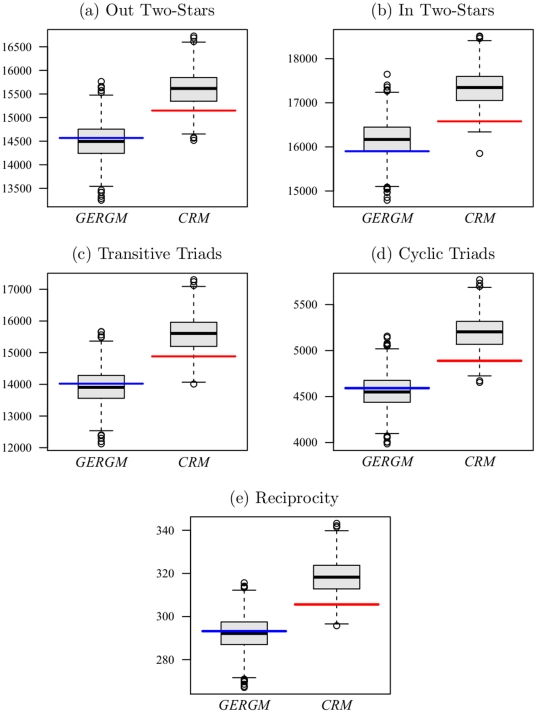
Dependence Feature Prediction. The boxplots represent the respective dependence statistic computed on 1,000 instances of the latent intensity network drawn from each model. Horizontal colored bars are placed at the statistic computed on the estimated intensity network.

The superior performance of the GERGM relative to the Cauchy regression is further depicted in Figure 7, which gives the predicted and observed network-level reciprocity and cycling measures from the GERGM and CRM. This figure shows that the regression does not adequately fit the dependencies (e.g. the lack of reciprocity) in the migration network. For example, it is theoretically expected that a network of change in migration would exhibit anti-reciprocity and anti-cycling. If a locale is experiencing a spike in migration to other places, that is likely indicative of some undesirable feature of said locale. This anti-reciprocal feature of the migration network cannot be integrated into the conventional regression modeling framework. Figure 7 serves as an additional test of the appropriateness of the independent regression model. If the CRM were the appropriate specification, the joint quantiles would be jointly uniform and these dependence statistics computed on the latent network would be predicted by the CRM. The GERGM accurately captures these features of the latent quantile network – with the observed value falling in the inter-quartile range of the values simulated from the GERGM.

This application shows the inability of the regression framework to model the sort of dependencies that we observe in real networks and the utility of having an inferential network model capable of accommodating networks with valued edges. In this case, we used our GERGM to produce insights into the migratory dynamics of the United States that could not have been produced otherwise.

## Discussion

The GERGM greatly expands the scope of networks that can be modeled within the ERGM framework. This is an important contribution for several reasons.

First, many networks have valued edges. We have examined one such network above, interstate migration in the U.S., but many others exist. For instance, the 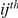 edge in the cosponsorship network in the U.S. Congress measures the number of bills Sponsored by 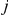 that are cosponsored by 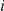 [15] in the two year period of the respective Congress. In previous research, [15] this network has been dichotomized to model with the ERGM. In a substantively much different application, [29] apply the ERGM to model a network created by dichotomizing pairwise correlations among the activity levels of 90 regions in the human brain. The direct analysis of a network of pairwise correlations could be conducted with the GERGM, without losing any information about the magnitude of the correlation, by using the simple transformation (i.e., 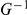) 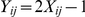.

Second, our method allows a researcher, who is not necessarily substantively interested in the interdependencies in the network, to test the restriction that the dependence parameters are equal to zero, meaning that interdependencies in the network do not matter. Such tests may be conducted using simple and well known methods such as the likelihood ratio test and Wald test.

Third, many common models for independent data (i.e. regression models typically estimated by least squares and/or maximum likelihood) are nested within the GERGM. Thus, if the endogenous structure of the network does not exert an effect, the researcher is returned a model with results identical to those they would have obtained using a regression. This is convenient not only because those independence models are familiar to political scientists, but because researchers may be dubious about the role of endogenous effects, but not want to risk model misspecification by ignoring them.

Lastly, and probably most importantly, the GERGM expands the set of substantive theories that researchers are able to evaluate empirically. For example, in our application, we gained insight into migration processes that would not have been possible absent the GERGM technology. This not only offers the opportunity to make progress on existing debates in the literature, but presents new theoretical horizons for scholars using relational data.

## References

[1] Cerami E, Demir E, Schultz N, Taylor BS, Sander C (2010). Automated network analysis identifies core pathways in glioblastoma.. PLoS ONE.

[2] Madi A, Kenett DY, Bransburg-Zabary S, Merbl Y, Quintana FJ (2011). Network theory analysis of antibody-antigen reactivity data: The immune trees at birth and adulthood.. PLoS ONE.

[3] Vass JK, Higham DJ, Mudaliar MAV, Mao X, Crowther DJ (2011). Discretization provides a conceptually simple tool to build expression networks.. PLoS ONE.

[4] Karrer B, Newman MEJ (2010). Stochastic blockmodels and community structure in networks.. Phys Rev E.

[5] Karrer B, Newman M (2009). Random graph models for directed acyclic networks.. Phys Rev E.

[6] Newman M (2009). Random graphs with clustering.. Phys Rev Lett.

[7] Garlaschelli D, Loffredo MI (2004). Fitness-dependent topological properties of the world trade web.. Phys Rev Lett.

[8] Bianconi G, Barabási AL (2001). Bose-einstein condensation in complex networks.. Phys Rev Lett.

[9] Myers S, Leskovec J (2010). On the convexity of latent social network inference..

[10] Richters O, Peixoto TP (2011). Trust transitivity in social networks.. PLoS ONE.

[11] Zhang Y, Friend AJ, Traud AL, Porter MA, Fowler JH (2008). Community structure in congressional cosponsorship networks.. Physica A.

[12] Mucha PJ, Porter MA (2010). Communities in multislice voting networks.. Chaos.

[13] Mucha PJ, Richardson T, Kevin M, A PM, Onnela JP (2010). Community structure in timedependent, multiscale, and multiplex networks.. Science.

[14] Butts CT (2008). A relational event framework for social action.. Sociological Methodology.

[15] Cranmer SJ, Desmarais BA (2011). Inferential network analysis with exponential random graph models.. Political Analysis.

[16] Cranmer SJ, Desmarais BA, Menninga EJ (2012). Complex dependencies in the alliance network.. Conict Management and Peace Science.

[17] Holland PW, Leinhardt S (1981). An exponential family of probability distributions for directed graphs.. J Am Stat Assoc.

[18] Berg J, Lässig M (2002). Correlated random networks.. Phys Rev Lett.

[19] Park J, Newman MEJ (2004). Statistical mechanics of networks.. Phys Rev E.

[20] Robins G, Snijders T, Wasserman S (1999). Logit models and logistic regressions for social networks: III. valued relations.. Psychometrica.

[21] Wyatt D, Choudhury T, Bilmes J (2010). Discovering long range properties of social networks with multi-valued time-inhomogeneous models..

[22] Villani M, Barbieri A, Serra R (2011). A dynamical model of genetic networks for cell differentiation.. PLoS ONE.

[23] Lee SH, Kim PJ, Ahn YY, Jeong H (2010). Googling social interactions: Web search engine based social network construction.. PLoS ONE.

[24] Wasserman S, Pattison P (1996). Logit models and logistic regressions for social networks: I. an introduction to markov graphs and *p**.. Psychometrika.

[25] Park J, Newman MEJ (2004). Solution of the two-star model of a network.. Phys Rev E.

[26] Genest C, MacKay J (1986). The joy of copulas: Bivariate distributions with uniform marginals.. The American Statistician.

[27] Zhang B, Horvath S (2005). A general framework for weighted gene co-expression network analysis.. Statistical Applications in Genetics and Molecular Biology.

[28] Kenett DY, Tumminello M, Madi A, Gur-Gershgoren G, Mantegna RN (2010). Dominating clasp of the financial sector revealed by partial correlation analysis of the stock market.. PLoS ONE.

[29] Simpson SL, Hayasaka S, Laurienti PJ (2011). Exponential random graph modeling for complex brain networks.. PLoS ONE.

[30] Casella G, Berger RL (2001). Statistical Inference.

[31] Stasinopoulos DM, Rigby RA (2007). Generalized additive models for location scale and shape (gamlss) in r.. Journal of Statistical Software.

[32] Krivitsky PN (2011). Exponential-family random graph models for valued networks.. arXiv.

[33] Geyer CJ, Thompson EA (1992). Constrained monte carlo maximum likelihood for dependent data.. Journal of the Royal Statistical Society Series B (Methodological).

[34] Wani JK (1968). On the linear exponential family.. Mathematical Proceedings of the Cambridge Philosophical Society.

[35] Snijders T (2002). Markov chain Monte Carlo estimation of exponential random graph models.. Journal of Social Structure.

[36] Handcock MS (2003). Assessing degeneracy in statistical models of social networks..

[37] Rinaldo A, Fienberg SE, Zhou Y (2009). On the geometry of discrete exponential families with application to exponential random graph models.. Electronic Journal of Statistics.

[38] Frank O, Strauss D (1986). Markov graphs.. Journal of the American Statistical Association.

[39] Snijders TAB, Pattison PE, Robins GL, Handcock MS (2006). New specifications for exponential random graph models.. Sociological Methodology.

[40] Handcock MS, Hunter DR, Butts CT, Goodreau SM, Morris M (2008). statnet: Software tools for the representation, visualization, analysis and simulation of network data.. Journal of Statistical Software.

[41] Geweke J, Dawid A, Berger J (1992). Evaluating the Accuracy of Sampling-Based Approaches to the Calculation of Posterior Moments.. Bayesian Statistics.

[42] Gill J (2008). Bayesian Methods: A Social and Behavioral Sciences Approach.

[43] Gelman A, Rubin DB (1992). Inference from iterative simulation using multiple sequences.. Statistical Science.

[44] Gelman A, Carlin JB, Stern HS, Rubin DB (2004). Bayesian Data Analysis.

[45] Clark GL, Ballard KP (1981). The demand and supply of labor and interstate relative wages: An empirical analysis.. Economic Geography.

[46] Levine PB, Zimmerman DJ (1999). An empirical analysis of the welfare magnet debate using the nlsy.. Journal of Population Economics.

[47] Preuhs RR (1999). State policy components of interstate migration in the united states.. Political Research Quarterly.

[48] Gimpel JG, Schuknecht JE (2001). Interstate migration and electoral politics.. The Journal of Politics.

[49] Chun Y (2008). Modeling network autocorrelation within migration ows by eigenvector spatial filtering.. Journal of Geographic Systems.

[50] Ke J, Chen X, Lin Z, Zheng Y, Lu W (2006). Kinetics of migration-driven aggregation processes on scale-free networks.. Phys Rev E.

[51] Ke J, Lin Z, Zheng Y, Chen X, Lu W (2006). Migration-driven aggregate growth on scale-free networks.. Phys Rev Lett.

[52] Mizera I, Mller CH (2002). Breakdown points of cauchy regression-scale estimators.. Statistics & Probability Letters.

[53] Jian L, MacKie-Mason JK (2008). Why share in peer-to-peer networks?. Proceedings of the 10th international conference on Electronic commerce.

[54] Opsahl T, Panzarasa P (2009). Clustering in weighted networks.. Social Networks.

[55] Saramäki J, Kivelä M, Onnela JP, Kaski K, Kertész J (2007). Generalizations of the clustering coefficient to weighted complex networks.. Phys Rev E.

[56] Gelman A, Gilks WR, Richardson S, Spiegelhalter DJ (1996). Inference and Monitoring Convergence.. Markov chain Monte Carlo in practice.

[57] Plummer M, Best N, Cowles K, Vines K (2010). coda: Output analysis and diagnostics for MCMC.. http://CRAN.R-project.org/package=coda.

